# Pediatric diffuse midline glioma: Understanding the mechanisms and assessing the next generation of personalized therapeutics

**DOI:** 10.1093/noajnl/vdad040

**Published:** 2023-04-12

**Authors:** Nicolina Jovanovich, Ahmed Habib, Jeffery Head, Farrukh Hameed, Sameer Agnihotri, Pascal O Zinn

**Affiliations:** Hillman Cancer Center, University of Pittsburgh Medical Center, Pittsburgh, Pennsylvania, USA; Department of Neurosurgery, University of Pittsburgh Medical Center, Pittsburgh, Pennsylvania, USA; Hillman Cancer Center, University of Pittsburgh Medical Center, Pittsburgh, Pennsylvania, USA; Department of Neurosurgery, University of Pittsburgh Medical Center, Pittsburgh, Pennsylvania, USA; Department of Neurosurgery, University of Pittsburgh Medical Center, Pittsburgh, Pennsylvania, USA; Hillman Cancer Center, University of Pittsburgh Medical Center, Pittsburgh, Pennsylvania, USA; Department of Neurosurgery, University of Pittsburgh Medical Center, Pittsburgh, Pennsylvania, USA; Department of Neurosurgery, University of Pittsburgh Medical Center, Pittsburgh, Pennsylvania, USA; Hillman Cancer Center, University of Pittsburgh Medical Center, Pittsburgh, Pennsylvania, USA

**Keywords:** Pontine, glioma, therapeutics, model, pediatric

## Abstract

Diffuse midline glioma (DMG) is a pediatric cancer that originates in the midline structures of the brain. Prognosis of DMG patients remains poor due to the infiltrative nature of these tumors and the protection they receive from systemically delivered therapeutics via an intact blood–brain barrier (BBB), making treatment difficult. While the cell of origin remains disputed, it is believed to reside in the ventral pons. Recent research has pointed toward epigenetic dysregulation inducing an OPC-like transcriptomic signature in DMG cells. This epigenetic dysregulation is typically caused by a mutation (K27M) in one of two histone genes—*H3F3A* or *HIST1H3B* –and can lead to a differentiation block that increases these cells oncogenic potential. Standard treatment with radiation is not sufficient at overcoming the aggressivity of this cancer and only confers a survival benefit of a few months, and thus, discovery of new therapeutics is of utmost importance. In this review, we discuss the cell of origin of DMGs, as well as the underlying molecular mechanisms that contribute to their aggressivity and resistance to treatment. Additionally, we outline the current standard of care for DMG patients and the potential future therapeutics for this cancer that are currently being tested in preclinical and clinical trials.

**Key Points**

The cell of origin of DMG is still controversial. Different oncogenic driver mutations uniquely affect the pathogenesis of DMG. Experimental therapeutics for DMG exploits its’ molecular vulnerabilities.

Diffuse midline glioma (DMG) is a highly aggressive pediatric cancer that originates in the midline structures of the brain.^1–3^ Approximately 10–15% of childhood brain tumors are DMGs, with 200–400 children in the United States being diagnosed each year.^1,4^ These tumors are the leading cause of brain tumor-related death in children, with the median survival time for those diagnosed being 9–11 months.^1,5,6^ Due to these tumors location in midline structures, as well as their infiltrative, diffuse nature, they are usually not candidates for surgical resection.^7^ This and the historical ineffectiveness of chemotherapeutics in treating DMG has centered the standard of care for these tumors around radiation therapy, with patients receiving 54–60 Gy doses over 6 weeks.^8–10^

In the past, a lack of viable biopsy tissue for ex vivo models was the main hindrance to progressing our understanding and treatment of DMG.^11–13^ However, even with the advent of new methods of biopsying tissue and collecting postmortem samples, and subsequently, the creation of more advanced models, there are still many challenges inherent to these tumors that has made advancements in their treatment arduous.^14,15^ Some of these challenges include their extensive inter-tumoral heteroegentiy, their diffuse nature, and the maintenance of an intact blood–brain barrier (BBB) that hinders delivery of systemic therapeutics to them.^16,17^

This review aims to describe our current understanding of DMG and the potential future therapies that could improve patient survival.

## Classification

**Figure 1.**
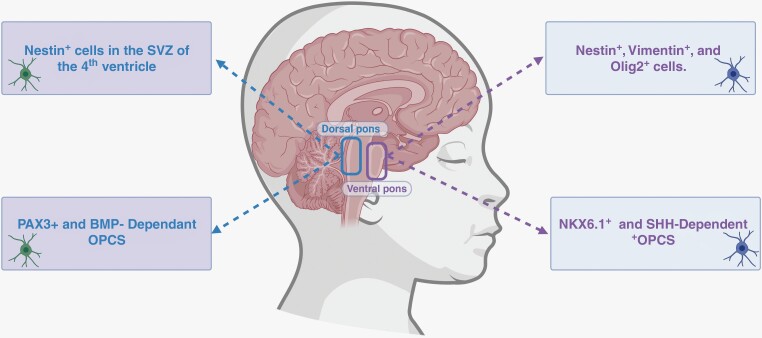
Graphical depiction of the anatomical location of the potential cell(s) of origin of diffuse midline gliomas: Purple box represents approximate anatomical location of Nestin+, Vimentin+, Olig2 + cells and NXK6-1+/ SHH-dependent OPCs and the blue box represents approximate anatomical location of Nestin + cells in the SVZ of the fourth ventricle and PAX3+/ BMP- dependent OPCs, all of which are potential cells of origin for DMGs.

Up until 2016, diffuse neoplasms within the pons were classified as diffuse intrinsic pontine glioma (DIPG) based off of their *H3F3A and HIST1H3B* histone mutations, as well as their radiological characteristics.^18^ Availability of increased samples for molecular analysis has since allowed for more precise distinction of these tumors; discovery of various, diffuse tumor types with these histone mutations outside of those originiating in the pons led the World Health Organization (WHO) to re-classify these tumors collectively as diffuse midline glioma, H3 K27M-mutant in 2016.^18,19^ Further characterization of the molecular pathways that drive oncogenesis in DMG tumors led to another re-classification of these gliomas to diffuse midline glioma, H3K27-altered to recognize the diverse set of mechanisms by which the epigenetics of these tumors can be altered via this pathway^19,20^ (Figure 1).

## Diffuse Midline Glioma Cell of Origin

Many studies in the past two decades have been aimed at identifying the precursor population of DMGs.^2,21^ In 2011, Monje et al. found Nestin-positive populations in the floor of the fourth ventricle and the ventral pons, either of which that could serve as the origin of DMGs (Figure 1). Ventral pons Nestin + cells were shown to co-express Vimentin, while fourth ventricle Nestin + cells did not. About half of the Nestin+/Vimentin + cells also expressed the transcription factor Olig2, a marker that typically specifies oligodendrocyte fate.^2^ The density of these Nestin + cells in the brainstem was tracked throughout childhood, and it was found that Nestin + cells were present in all ventral brainstem structures until about age 2, when they waned. In the pons, these Nestin + cells re-increased in density in middle childhood; This resurgence corresponds with and could explain the predominance of DMGs in children aged 5–9 years old.^2^

Due to the expression of Olig2 + in the Nestin + population proposed by Monje et al., oligodendrocyte-progenitor cells (OPC) have also been looked at as a possible precursor cell of DMG.^22,23^ Fortin et al. recently used an Olig2^Cre^ system to target an Activin A Receptor Type 1 (ACVR1) mutated protein (ACVR1^floxG328V/+^), which is commonly found in the H3.1K27M subset of DMGs, to OPCs in a murine model. ACVR1^floxG328V/+^; Olig2^Cre^ cells with a reporter allele, ROSA26^LSL-tdTomato^, were shown to have a 2-fold increase in proliferation when compared to controls, which led to oligodendrocyte expansion and neurological symptoms in mice. The addition of Hist1h3b^K27M/+;^Pik3ca^floxH1047R/+^ mutations to ACVR1^floxG328V/+^; Olig2^Cre^ cells induced tumors consistent with high-grade diffuse gliomas, suggesting that OPCs could be the tumorigenic origin cell of DMG tumors.^22^ Another study by Anderson et al. confirmed the tumorigenic potential of Olig2 + cells by showing that DMG cells with Olig2 expression formed Olig2 + brainstem gliomas with 100% penetrance in xenograft models. Such strong evidence suggests the role of Olig2 + as an onco-requisite factor in DMG.^23^

Since, evaluation of DMG tumors via single-cell RNA sequencing has shown H3K27M cells to have transcriptomic signatures most closely matched to OPCs.^24^ Additionally, the congruence between chromatin and transcriptome defined states of DMG tumor samples has revealed cell-state-specific genes linked with predictive chromatin (GPCs) that are commonly enriched in OPC/OC like cells, supporting the role of OPCs as the cell of origin in DMG tumors.^25^ A more recent study by Jessa et al. further showed that distinct OPC cell niches may give rise to molecularly distinct midline gliomas.^26^ Cluster configuration of the *HOXA5*, *HOXB4*, and *HOXD8* genes and activation of the transcription factor NKX6-1in H3.1K27M DMG cells points toward NKX6-1^+^/SHH-dependent OPCs in the ventral pons as these tumors’ cell of origin. Contrastingly, the cluster configuration of the *HOXB4* and *HOXD4* genes and the activation of PAX3 + in H3.3K27M DMG cells points toward PAX3+/BMP-dependent OPCs in the dorsal pons as the origin of these tumors.^26^

Other papers have proposed NSCs as the cell of origin for DMGs. In a recent paper, Haag et al. created a Cre-conditional K27M mutation in both induced neural stem cells (iNSCs) and iOPCs. To test the oncogenicity of iOPCs and iNSCs, WT or K27M iOPCs/iNSCs with and without TP53 knockdown were injected into the brainstems of mice. They found that only H3.3K27M; TP53^-/-^ iNSC cells produced tumors. This failure of iOPCs to produce tumors suggests that these cells may not be the true cell of origin for DMGs. Haag et al. went on to speculate that Olig2 + expression in DMG tumors may not be the consequence of an OPC cell of origin, but instead, that it may be a consequence of H3K27M-induced epigenetic dysregulation; This dysregulation is marked by hypomethylation of promoters controlling OPC gene expression and can cause subsequent enrichment of these genes in DMG cells, leading to a pseudo-OPC transcriptomic signature.^27^ Another study looking at potential cells of origin showed that when H3K27M is over-expressed in NSC and OPC driven models of DMG, this oncogenic driver only impacts tumor latency and proliferation in NSC models, further supporting NSCs as DMGs’ cell of origin.^28^

## Pathogenesis and Tumorigenic Mechanisms of DMG

### H3K27M

DMG tumor analyses over the past few decades have shown that a point mutation, K27M, in the histone genes *H3F3A* and *HIST1H3B* are the primary oncogenic drivers of this pediatric cancer (Table 1).^29^ Histones are positively charged proteins that structurally support and package chromosomal DNA.^30^ They can be transcriptionally modified by the addition of acetyl and methyl groups to lysine residues within the histone, which increase and decrease the propensity for gene transcription, respectively.^31^ Substitution of lysine for methionine at the 27th position of the H3 histone protein results in an inability for the polycomb repressive complex 2 (PRC2) to methylate, and thus, epigenetically control, these histone proteins and the DNA they are associated with.^32^

**Table 1.** Common Genes with Oncogenic Driver Mutations in Diffuse Midline Glioma Tumors

Gene	Incidence (%)	Protein	Oncogenic Mechanism
*H3F3A*	~65%	Histone; structurally supports and packages DNA	Inability of polycomb repressive complex 2 (PRC2) to bind to lysine residue and methylate, leading to epigenetic dysregulation of DMG cells; forms H3K27M-K27ac heterotypic nucleosomes
*HIST1H3B*	~12–18%	Histone; structurally supports and packages DNA	Inability of polycomb repressive complex 2 (PRC2) to bind to lysine residue and methylate, leading to epigenetic dysregulation of DMG cells; form H3K27M-K27ac heterotypic nucleosomes
*EZHIP*	~10–15%	EZH2 inhibitor; mimics H3K27M oncohistone	Binds to PRC2 and inhibits it via C-terminal peptide that mimics H3K27M oncohistone, leading to epigenetic dysregulation of DMG cells
*TP53*	~60–80%	Tumor suppressor that regulates the G1/S checkpoint during cell division	Allows cells to bypass G1/S checkpoint, and thus, promotes stemness and proliferation of DMG cells
*ACVR1*	~32% (87% of these being H3.1K27M DMGs)	Involved in nervous system development and regulation	Activates mesenchymal gene programs and promotes stemness, de-differentiation, and proliferation of DMG cells
*BMI1 Proto-Oncogene, Polycomb Ring Finger*	unknown	Subunit of the polycomb repressive complex 1 (PRC1) that modifies histones and controls chromatin access	Helps to maintain differentiation block in DMG cells, possibly through binding to transcription start sites of genes important for diferentiation

**Figure 2.**
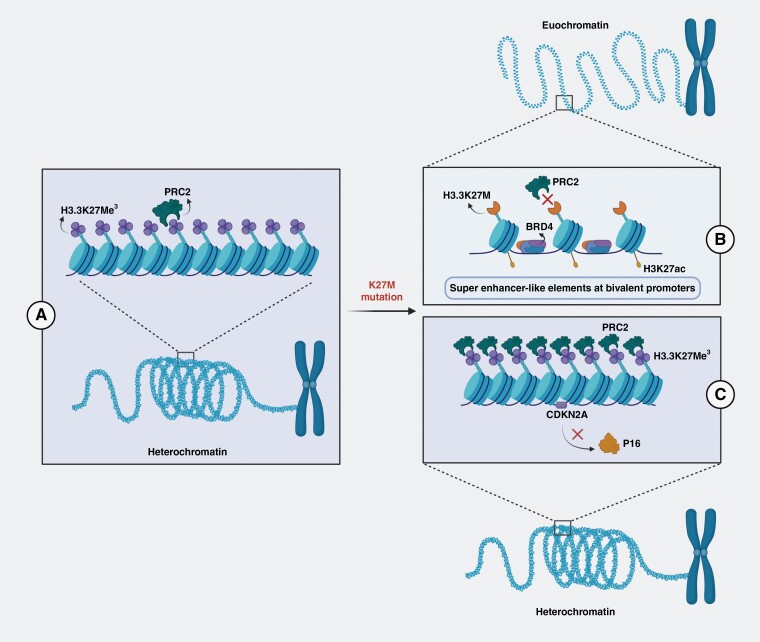
Graphical depiction of the epigenetic dysregulation in the genome of diffuse midline glioma tumors with K27M mutations: (A) Methylation of lysines at the 27th amino acid position on histones results in condensation of DNA into heterochromatin, preventing transcription. (B) H3.3K27M mutation excludes PRC2 from areas of chromatin co-localized with K27-ac sites, allowing bromodomain proteins to bind and help form super-enhancer-like elements that allow for high, un-checked levels of gene expression. C) PRC2 preferentially methylates certain histones, silencing genes, such as *CDKN2A,* that encode tumor suppressor proteins, resulting in further oncogenic transformation and uncontrolled cell proliferation.

Although it was originally believed that the H3K27M mutation caused sequestration of PRC2, one of the complexes responsible for histone methylation, recent studies have suggested that this premonition is incorrect.^32,33^ Piunti et al.’s genome-wide distribution analysis of H3K27me,^3^ H3.3K27M, and PRC2 in DMG showed that PRC2, and its subunits, were present throughout the genome but excluded from chromatin containing H3K27M-K27ac heterotypic nucleosomes^33^ (Figure 2). Consequently, PRC2’s activity is not affected significantly outside of these K27M containing sites, as evidenced by H3K27me^3^ marks in other parts of the genome of DMG cells. In fact, multiple studies have shown that the continued catalytic activity of the PRC2 complex is essential to maintaining DMG proliferative ability via repressing neuronal differentiation and various cell cycle checkpoint inhibitors, such as p14 and p16.^33–35^ In studies where DMG cells were treated with PRC2 subunit inhibitors, their ability to form colonies was significantly reduced and the transcriptional expression of p16 was sometimes rescued.^33–35^

H3.3 histones are localized at K27-ac sites where bromodomain proteins bind and help to form super-enhancer-like elements.^33^ These elements are highly implicated in the aggressivity of various cancers.^36,37^ In Piunti et al.’s study, treatment of DMG cell cultures with JQ1, a bromodomain and external-domain (BET) inhibitor, resulted in a decrease in the proliferative capacity of and a neuron-like morphological change in these cells. This morphological change was accompanied by an upregulation in neuronal markers, such as tubulin beta class III (TUBB3) and microtubule associate protein 2 (MAP2), and suggests these super-enhancer like elements may block DMG cells from differentiating.^33^ A recent study by Haag et al. confirmed this when it showed that expression of the H3.3K27M mutation in iNSCs increased the transcription of genes related to stemness, such as Nestin and neuron-glial antigen 2 (NG2).^27^

H3.3K27M DMGs, which make up over 70% of histone-mutant DMGs, and H3.1K27M DMGs drive different oncogenic signatures, with H3.3K27M DMGs showing a proneural/OPC signature with up-regulated expression of pro-metastatic gene sets and H3.1K27M DMGs showing a mesenchymal/astrocytic signature with up-regulated expression of pro-angiogenic/hypoxic gene sets.^38^ These key phenotypic differences, including association of these distinct mutations with different co-oncogenic drivers (H3.3 with p53 and platelet-derived growth factor alpha [PDGFRA] and H3.1 with ACVR1), lead to a difference in clinical outcomes of these patients^10,38,39^; Pediatric H3.3K27M DMG patients have been shown to have a worse response to radiotherapy (55.3% vs 85%, *P* = .263) and worse overall survival (9.2 months vs 15 months, *P* = 4.51e − 05; 10.1 months vs 14.2 months, *P* < .001) when compared with pediatric H3.1K27M DMG patients.^38,39^

### Enhancer of Zeste Homologs Inhibitroy Protein (EZHIP)

H3WT tumors are found in 10–15% of DMG patients and tend to overexpress the enhancer of zeste homologs inhibitory protein (EZHIP), a protein thought to be responsible for these tumors methylation dysregulation.^40,41^ EZHIP contains a conserved C-terminal peptide that mimics the amino acid sequence of the H3K27M oncohistone, allowing it to bind to the active site of PRC2 and alter its conformation, resulting in inhibition.^42^ A study by Jain et al. has suggested that allosterically activated PRC2s are more susceptible to inhibition by EZHIP, which could explain the loss of H3K27me^3^ spread in tumors.^43^ Despite having an intact histone protein, these tumors cluster close to H3K27M DMG’s upon methylation analysis.^41^ Five-year survival time of patients with H3WT DMGs has been shown to be significantly higher in comparison to patients with H3K27M DMGs (68.8% vs 6.3%; *P* = .0002).^44^

### Tumor Protein P53

Mutations in the tumor protein P53 (*TP53*) gene which encodes p53—a tumor suppressor that regulates the G1/S checkpoint during cell division—are found in about 70-80% of H3K27M DMGs.^28,45,46^ H3K27M cells are non-viable when the histone mutation is the sole oncogenic driver, and thus, an additional mutation is required to restore viability and promote cancerignesis.^22,27,28,47^ In a recent study, sole expression of H3K27M in IPSCs led to cell death, but adding TP53 loss largely rescued the H3K27M cells and helped to drive gliomagenesis.^27^

This survival benefit conferred by TP53 mutations has also been shown to translate to clinical radioresistance.^46,48^ In a study by Werbrouck et al., genetic profiles of GSCs with K27M mutations were evaluated for their tolerance to irradiation. While there was no correlation between radioresistance and the type of histone mutation, there was a significant correlation between TP53 mutation status and radioresistance. DMG cells without TP53 mutations had an LD50 average of 1.1 Gy ± 0.5, while cells with a TP53 mutation had an LD50 average of 5.5 Gy ± 1.7 (*P*-value = .0001). These radioresistance measurements described in vitro translate to radioresistance in patients, as TP53 mutations were found to be negatively correlated with time to first progression post-radiotherapy.^49^ Moving forward, genetic analysis of DMG stereotactic biopsies could be extremely important in determining a patient’s predicted response to RT and aid future providers in constructing an effective treatment regimen.

### Activin A Receptor Type 1 (ACVR1)

Mutations in the *ACVR1* gene, which translates into a protein involved in nervous system development and regulation, occur in about 32% of DMG patients and commonly co-associate with H3.1K27M mutations(87% of mutation co-associate with H3.1K27M).^40,50–52^ To elucidate the role ACVR1 plays in promoting oncogenesis in DMG precursor cells, Hoeman et al. monitored the changes in gene expression associated with ACVR1^R206H^ expression. Gene set enrichment analysis showed increased expression of *CD44*, *tenascin C* (TNC), and *SNAIL2* genes, suggesting the activation of mesenchymal gene programs by mutant ACVR1. Additionally, epithelial to mesenchymal transition genes and IL-6/JAK/STAT3 signaling pathway genes were enriched. These results suggest that ACVR1 mutations may activate an epithelial to mesenchymal transition that promotes stemness, de-differentiation, and aggressive proliferation of DMG cells.^51^

### BMI1 Proto-Oncogene, Polycomb Ring Finger

Transcriptome analysis of DMG cell samples have shown that BMI1, a subunit of the polycomb repressive complex 1 (PRC1), mRNA is significantly elevated in these cancerous cells when compared to cells of normal brain tissue. Targeting of BMI1 with shRNAs in DMG neurospheres has shown that BMI1 plays a role in maintaining a differentiation block in these cancerous cells, possibly through binding to the transcription start sites of genes important for cell differentiation.^53^

## Current Treatment Modalities for DMG

Ineligibility for resection, therapeutic challenges posed by an in tact BBB, and heterogeneity of these tumors pose extensive challenges to treating DMG patients. While chemotherapy is sometimes included in the regimen of these patients, the current standard of care centers on fractionated radiation therapy, which is given in 30–33 fractions at 1.8 Gy daily.^8,12,49,51,54^ Radiation treatment does provide some temporary relief; however, it confers a survival benefit of only a few months, during which the quality of patients’ lives is very poor.^12^

## Therapeutic Modalities in Preclinical/Clinical Trials

Novel treatments that are more cytotoxic to DMG cells, and consequently, that could improve the overall survival (OS) and quality of life of patients past the current standard of care, are currently being tested in preclinical models and human clinical trials around the world (Table 2).

**Table 2.** Current and Experimental Therapeutics Used to Treat Diffuse Midline Glioma Patients

Therapy	Mechanism of Action	Dose	Current Therapeutic vs. Experimental Therapeutic	Clinical Trial	Important Findings
Current Therapies					
Fractionated external beam radiation therapy	Induces ionization that can act on cellular molecules and cause DNA damage	1.8Gy over 30 fractions (total of 54Gy)	Current Therapeutic	N/A	N/A
Chemotherapy	Varies; Acts on cells that are dividing and disrupts replication/division	Varies	Current Therapeutic & Experimental Therapeutic	NCT02992015, NCT05413304	N/A
Therapies in Preclinical Trials					
PRC2 Inhibitors	Inhibits chromatin-methylating enzyme important for epigenetic regulation	varies	Experimental Therapeutic	none	Treatment of DMG cell lines with PRC2 inhibitors led to reduced proliferation of these cells in culture.
CDK4/6 Inhibitors	Inhibits enzyme that promotes cell entrance into S phase	varies	Experimental Therapeutic	NCT05413304	DMG cells treated with CDK4/6 inhibitors in vitro showed reduced proliferation. Additionally, treatment of DMG mouse models with these inhibitors resulted in prolonged survival.
BMI1 Inhibitors	Inhibits subunit of PRC1 which is essential to the formation of the central spindle during cell division	varies	Experimental Therapeutic	NCT03605550	DMG mice treated with BMI1 inhibitors showed prolonged survival. Concurrent treatment with a BMI1 inhibitor and a BH3 mimetic led to a sustained anti-tumor response, even one week after therapy was ended.
Therapies in Clinical Trials					
ONC201	Dopamine D2 receptor (DRD2) andtagonsit and caseinolytic protease proteolytic subunit (ClpP) agonist	varies	Experimental Therapeutic	NCT03416530, NCT05009992, NCT05476939, NCT02525692	Treatment with ONC201 in nano- to micromolar ranges results in decreased DMG cell viability. Clinical trials have revealed no dose-limiting toxicities. On one trial, two pediatric patients have remained progression-free for > 53 weeks.
CAR T Cell Therapy	T cell targeting of antigens expressed on DMG tumors	varies	Experimental Therapeutic	NCT04185038, NCT05478837, NCT04196413,	DMG patients treated with GD2-CAR T cells showed some radiographic improvement and did not exhibit any side effects above Grade III. DMG patients treated with B7-H3 CAR T-cells have exhibited stable disease post-infusion and no side effects above Grade III.
Chemotherapeutics	Varies; Acts on cells that are dividing and disrupts replication/division	varies	Experimental Therapeutic	NCT02992015, NCT05413304	Convection enhanced delivery (CED) of carboplatin into DMG tumor cavities has been shown to cause minimal side effects. Ten out of the thirteen patients treated with CED carboplatin and sodium valproate had their DMG controlled to the pons. Progression free survival and overall survival for these DMG patients were 13 and 15 months respectively, compared to 6 and 11 months seen with radiation therapy alone.
[^124^I]-8H9	Radiolabeled-antibody targeting the diffuse midline glioma antigen B7-H3	0.24 to 4.4 mL	Experimental Therapeutic	NCT01502917	Convection enhanced delivery (CED) was used to deliver [^s^I]-8H9 into the tumor cavity of DMG patients. PET showed localization of this therapeutic to the brainstem tumors. Median survival of patients in this trial was 15.3 months, which was higher than that of radiation alone (11 months).

### Preclinical Trials

#### Polycomb repressive complex 2 (PRC2) inhibitors

Polycomb repressive complex 2 (PRC2) is a chromatin-modifying enzyme that methylates the lysine at position 27 on histone H3.^55^ H3K27M mutations have a dominant negative effect on EZH2, the active catalytic component of the PRC2 complex, resulting in a reduction of a repressive H3K27me3 gene mark.^56^ Despite this, there are still some gene loci with residual H3K27me3 deposition, including the *CDKN2A* locus, which encodes for two tumor suppressor genes: INK4a (p16) and ARF (p14).^33,57^ Consequently, the PRC2 complex has become a target of interest for DMG therapeutics.

Mohammad et al. tested the effect of a PCR2 complex subunit (EZH2) inhibitor on 6 DMG cell lines, as well as a pediatric GBM cell line with a wild-type H3 and a GBM cell line with a G34R mutation in H3.3. All of the primary DMG cell lines showed reduced proliferation and decreased methylation at the *CDKN2A* locus in response to EZH2 inhibition. Neither of the GBM cell lines had reduced colony formation, suggesting that this therapeutic effect is specific to DMG and the epigenetics that drive it.^34^ A similar anti-proliferative effect was seen in DMG cells subjected to EED and SUZ12 inhibition (PRC2 subunits) in a study done by Piunti et al.^33^ Given that this therapeutic has only been tested in preclinical models, clinical trials would have to be conducted to determine its efficacy in DMG patients.

#### Cyclin D Cdk4,6 (CDK4/6) inhibitors

Cyclin D-Cdk4,6 (CDK4/6) is an enzyme that promotes cell proliferation and entrance into S phase via phosphorylation of substrate proteins.^58^ It is inhibited endogenously by p16. Because p16 expression has been found to be suppressed in DMGs, CDK 4/6 inhibitors have become a therapy of interest in DMG patients.^33,34^

In vitro studies have shown that DMG cells are sensitive to CDK4/6 inhibition, with cells exhibiting reduced proliferation and G_1_ cell cycle arrest after treatment.^59,60^ When taken a step further and tested in a DMG mouse model, treatment with PD-0332991 (PD), a CDK4/6 inhibitor, also resulted in cell cycle arrest and significantly prolonged survival of DMG treated mice in comparison to DMG non-treated mice.^61^ A recent clinical trial [NCT05413304] has been initiated to determine if a regimen of pre-operative abemiciclib (CDK 4/6 inhibitor) and post-operative abemiciclib + temozolomide is effective in treating DMG patients.

#### BMI1 proto-oncogene, polycomb ring finger inhibitors

BMI1 is a subunit of the Polycomb Repressive Complex 1 (PRC1), which is important for gene regulation during development. It has been shown to play a role in the pathogenic differentiation block seen in DMG cells, making it a possible target for future treatment regimens.^53^

When tested in a DMG murine model, PCT028—a BMI1 inihibitor—prolonged survival of diseased mice. Transcriptome analysis showed treated mice had increased expression of tumor suppressor genes p16 and p21, as well as increased expression of a senescent-associated protein, Beta-gal. Mice treated with in combination with PCT028 and a BH3 mimetic, which antagonizes anrti-apoptitic proteins, had reduced tumor burden and continued to have minimal tumor burden even one week after treatment was stopped. Contrastingly, those treated with monotherapy saw tumor recurrence after therapeutic cessation.^53^ Success of these inhibitors in murine models has led to a clinical trial [NCT03605550] studying the therapeutic efficacy of a second-generation BMI1 inhibitor, PTC596, in treating patients with newly diagnosed DMG.

### Clinical Trials

#### ONC201

ONC201, a dopamine D2 receptor (DRD2) antagonist and caseinolytic protease proteolytic subunit (ClpP) agonist, has been shown to activate DR5/TRAIL-mediated apoptosis, as well as other anti-proliferative transcriptional programs, in tumor cells.^62,63^ The prognostic importance of ClpP in pediatric gliomas, as well as the ability of this drug to cross the intact BBB, has made it of therapeutic interest in DMG patients.^64–66^

In vitro assessment of ONC201 has shown that it reduces DMG cell viability in nano- to micromolar ranges.^64^ Treated cells exhibit higher levels of mitochondrial reactive oxygen species and show robust mitochondrial morphological changes, signifying mitochondrial shut down. Additionally, a reduced percentage of these cells are found to be a part of cycling and/or OPC-like subpopulations when compared to control DMG cells, confirming ONC201’s anti-tumorgenic effect.^64^

Various clinical trials are currently looking at the safety and efficacy of ONC201 in pediatric DMG patients [NCT03416530, NCT05009992, NCT05476939] in combination with radiotherapy and other biologics. When scaled to body weight, ONC201 has not exhibited any dose-limiting toxicities, with headache, nausea, and seventh cranial nerve disorder being the most common adverse events in treated DMG patients.^67,68^ Furthermore, in one clinical trial, two pediatric patients have remained on therapy and been progression free for > 53 weeks.^69^

#### Chimeric antigen receptor (CAR) T-cell therapy

**Figure 3.**
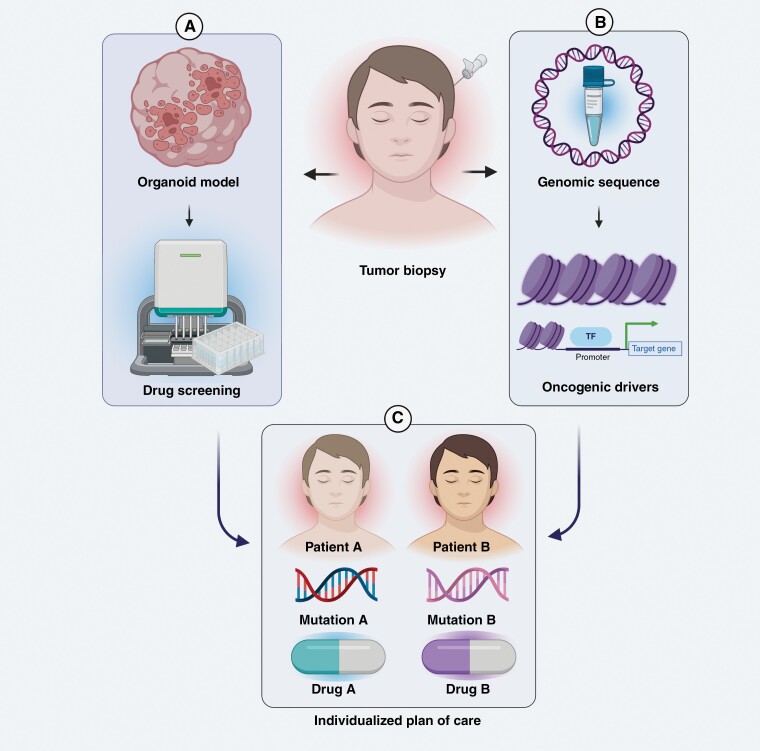
Graphical depiction of the use of a personalized model and techniques to treat diffuse midline glioma patients: (A) Biopsies of DMG tumors can be seeded into 3D-organoid models and tested against a panel of therapeutics in order to find the most efficacious therapy on a patient by patient basis. (B) Biopsies of DMG tumors can be genetically analyzed for certain oncogenic drivers that have known therapeutic sensitivities and vulnerabilities. (C) Patient A and Patient B receive personalized care plans that are optimized to treating their genetically distinct tumors.

The expression of a stable set of antigens across DMG tumors—most prominently a carbohydrate-containing sphingolipid, ganglioside 2 (GD2), and an immune checkpoint molecule, B7-H3—that can be targeted by chimeric antigen receptor (CAR) T-cell therapy, as well as the continued refinement of personalized medicine techniques (Figure 3), has made immunotherapy a promising prospective treatment for DMG patients.^70,71^

In 2018, Mount et al. tested the efficacy of GD2-CAR T cells in xenografted murine models. DMG xenograft mice displayed reduced tumor burden in response to treatment, with histological analysis showing that GD2 expressing DMG cells had largely been eradicated from these mice. In lieu of these results, a clinical trial was started to look at the cytotoxicity of GD2-CAR T cells against brain and spinal DMG tumors [NCT04196413]. Treatment with CAR T cells was supplemented with chemotherapy, and preliminary results have shown some radiographic improvement in patients. Notably, no patients have experienced side effects greater than Grade III classification, suggesting that this therapy has an acceptable safety profile.^71^ A clinical trial testing the efficacy of B7-H3 CAR T-cell locoregional therapy in treating DIPG/DMG and recurrent and refractory CNS tumors is also currently underway [NCT04185038].^72^ Preliminary data on two treated patients have revealed detectable B7-H3 CAR T cells in their cerebrospinal fluid. No side effects above grade III have been reported and both patients have been shown to have stable disease post-CAR T-cell infusion.^73^

#### Chemotherapeutic agents

The continued use of systemic chemotherapy in the treatment of DMG has not significantly improved the OS of these patients; This is believed to be in part due to the inability of chemotherapeutics to extravasate across the BBB and penetrate these tumors.^54,74^

The ability of gemcitabine to penetrate GBM xenograft tumors in cortical and pontine locations was tested in a study by Green et al. The ratio of the concentration of gemcitabine that penetrated tumor tissue compared to that in the normal brain was higher in cortical tumors than pons tumors, suggesting that chemotherapeutics are less able to breach the BBB in the pons.^17,54^

In response to these studies, a phase 0 clinical trial was initiated looking at the ability of i.v. gemcitabine to penetrate the tumors of DMG patients [NCT02992015]. Two patients were enrolled and biopsy cores of their tumors showed a mean gemcitabine concentration of 7.65 µM and 3.85 µM, respectively; This is comparable to the mean gemcitabine level in a clinical trial of adult GBMs (3.48 µM), suggesting that adequate drug penetrance of the tumor was achieved, and thus, other factors may play a role in the inefficacy of these drugs in treating DMG patients.^54^

Despite these results, researchers have begun to look at alternative methods for delivering chemotherapeutic agents, other than the systemic route, that may increase their concentration in these neoplasms. In one study, the efficacy of delivering carboplatin via convection-enhanced delivery—a new therapeutic strategy that delivers drugs directly into brain tumor cavities through a surgically placed catheter—was studied.^75,76^ A Renshaw Drug Delivery System was used for 7 cycles of 3–6 weekly pontine infusions of carboplatin (0.12–0.18 mg/mL) or sodium valproate (14.4–28.8 mg/mL) monotherapy, or combination therapy of both drugs. The infusions were safe and well-tolerated, with minor side effects well controlled with steroid administration. In 10 out of the 13 patients, treatment controlled DMG infiltration to the pons. The mean progression-free survival and overall survival of patients with CED carboplatin and sodium valproate therapy were 13 months and 15 months, respectively, which is longer than that seen with traditional radiotherapy (6 months and 11 months, respectively).^76^

#### [^124^I]-8H9

In addition to being used for carboplatin administration, CED has been used in a phase 1, single-arm, single-center, dose-escalation study [NCT01502917] to deliver a radiolabeled antibody, [^124^I]-8H9, into the brainstems of 28 DMG patients who had received external beam radiation at least 4 weeks before enrollment. Infusion volumes ranged from 0.24 mL to 4.4 mL. No Grade 4 adverse events or dose-limiting toxicities were observed. Thus, this therapy was deemed to have a good safety profile.^77^

PET scans of patients showed that [^124^I]-8H9 localized to the lesion in the brainstem soon after infusion and remained there until it was cleared (mean clearance time was 36 h for fast, and 59 h for slow). The mean lesion absorbed dose was measured to be 0.39 Gy/MBq^124^I, while the systemic absorbance was negligible (lesion to whole-body absorbed dose ratio was 1285). As of March 2018, the median overall survival of the 25 patients who were evaluable was 15.3 months, which is higher than that observed for radiotherapy alone (11 months). Median overall survival varied by dose level, with the middle dose level of 3 conferring the greates survival benefit (17.1 months).^77^

## Conclusions

Refinement of stereotactic biopsy techniques, as well as postmortem cell cultures, has allowed for ample collection of DMG patient tissue for use in biological models in recent years.^14,15^ Assembly and use of patient-derived in vitro and in vivo models has spurred advancements in discovering the possible cell of origin, pathological mechanisms, and therapeutic sensitivities of this pediatric brainstem cancer.^27,34,54,71,78^

While it is almost unanimously agreed upon that the DMG cell of origin resides in the ventral pons, the specific cell niche responsible for this cancer is still under investigation.^2,22,27^ Monje et al. originally proposed a Nestin+/Vimentin+/Olig2 + population as the precursor population for DMG; Since, multiple studies have confirmed the ability of various Nestin + and Olig2 + cell populations in the ventral pons and fourth ventricle to produce aggressive pontine tumors when engineered to express common oncogenic drivers of DMG.^12,21,22^ The commonality of the Olig2 signature originally led to the belief that the cell of origin is an OPC, with recent evidence supporting this role and further suggesting that distinct OPC niches may be responsible for molecularly distinct DMGs.^25,26^ However, conflicting evidence from Haag et al. suggests that the cell of origin may be an NSC that harbors a OPC-like transcriptomic signature brought about by epigenetic dysregulation of bivalent promoters in this disease, leaving the true cell of origin up for debate.^22,27^

The epigenetic dysregulation touched upon by Haag et al. has been found to be a significant underlying pathological mechanism of DMG.^27,33,34^ Although the histone mutation, H3K27M, was originally believed to sequester the PRC2 enzyme and inactivate it, recent studies have pointed to the exclusion of PRC2 from chromatin containing H3K27M-K27ac heterotypic nucleosomes, and furthermore, the necessity of its activity for the maintenance of a stem cell-like state in this cancer.^33,34^ The participation of H3K27M in super enhancer-like elements that reprogram the enhancer landscape, as well as the enrichment of PRC2 at tumor suppressor genes, results in a hallmark differentiation block and immortal proliferative cycle that drives the oncogenic nature of DMG-K27 altered cells.^33–35^

The current standard of care for DMG, which is centered around radiation therapy, has proven to be insufficient in significantly improving patient outcomes.^8^ Thus, various therapeutics engineered to target the underlying aberrant pathways in DMG cells have started to be tested in preclinical and clinical models. These include PRC2, BET1, and CDK4/6 inhibitors, as well as various immunotherapy agents (CAR T cells and CED of [^124^I]-8H9).^33,34,59,71,77^

While these trials have yielded promising results, the ability of systemic drugs to extravasate across the BBB still remains a major challenge in delivering these drugs to infiltrative tumors in a sufficient concentration.^17,54^ Convection enhanced delivery, in which drugs are delivered directly into the tumor cavity via a surgically placed shunt, bypasses the BBB and provides a promising means for concentrating therapies at highly cytotoxic doses in the core of these devastating tumors.^75^ Because gliomagenesis of DMG is driven by an epigenetic, differentiation block, CED administration of a drug that could reverse this block, in combination with a cytotoxic drug, could target this cancer from two biological angles and possibly provide superior clinical benefit to the current standard of care.

As the standard of care for DMG advances, it would make sense for care plans to shift toward personalized medicine. The continued improvement in our ability to genetically manipulate murine models via various molecular biology technologies has allowed for research on specific genetic, DMG variants and how these variants differentiate from each other in tumorigenic mechanisms, as well as therapeutic sensitivities. It has been found that various oncogenic profiles in DMG, such as cells with *TP53* mutations or cells with *INK4a-ARF* loss, are less sensitive to radiation and more sensitive to CDK4/6 inhibitors, respectively.^49,59^ The differential therapeutic response of DMG cells characterized by differential mutational burdens suggests that molecular characterization of patients’ tumors is needed to build individualized care plans. Such characterization could be done if samples could be seeded into complex, 3D organoid models, as well as murine models, and tested against drug panels; This personalized modeling and testing of DMG tumors could facilitate the development of a personalized plan of care for each patient, with the intent of significantly improving the quantity and quality of life of DMG patients past that of the current standard of care.
